# Semelil as Adjunctive Therapy in Chronic Periodontitis: A Preliminary Randomized Controlled Clinical Study

**DOI:** 10.22037/ijpr.2020.113604.14399

**Published:** 2021

**Authors:** Hoori Aslroosta, Mojgan Paknejad, Mohaddeseh Davari, Solmaz Akbari, Mina Taheri, Mohammad Abdollahi

**Affiliations:** a *Dental Research Center, Dentistry Research Institute, Tehran University of Medical Sciences, Tehran, Iran. *; b *Department of Oral and Maxillofacial Medicine, School of Dentistry, Semnan University of Medical Sciences, Semnan, Iran. *; c *Department of Periodontics, School of Dentistry, Tehran University of Medical Sciences, Tehran, Iran. *; d *Toxicology and Diseases Group, Pharmaceutical Sciences Research Center (PSRC), The Institute of Pharmaceutical Sciences (TIPS), and Department of Toxicology and Pharmacology, School of Pharmacy, Tehran University of Medical Sciences, Tehran, Iran.*

**Keywords:** ANGIPARS, Semelil, Oxidative stress, Periodontitis, Plant extracts

## Abstract

Host modulation therapy is recently employed to improve periodontal treatments outcome. This randomized controlled clinical trial aimed to evaluate the effects of Semelil (ANGIPARS) as an adjunct to non-surgical treatment in patients with chronic periodontitis. Forty-four healthy subjects with moderate to severe chronic periodontitis were enrolled in the study. After completion of phase I periodontal therapy, including oral hygiene instruction, scaling, and root planing, the patients were randomly divided into two groups to receive capsules of Semelil (test) or placebo (control), consuming two capsules a day for three months. Clinical parameters (probing depth [PD], clinical attachment level [CAL], modified sulcular bleeding index [MSBI], modified gingival index [MGI], and plaque index [PI]) and biochemical parameters (interleukin-1β [IL-1β], 8-hydroxy-2-deoxyguanosine [8-OHdG]), and lipid peroxidation [LPO]) were measured at baseline and after completion of treatment. Twenty-five patients completed the study: 15 in the test group and 10 in the control group. All clinical and biochemical parameters were significantly improved from baseline to the final measurements in both groups (*p < *0.001). The changes were more pronounced in the test group in comparison to the control group. However, the differences between the groups were significant only for MGI, MSBI, PD, and CAL (*p < *0.05). Semelil may reveal promising results as an adjunctive treatment for chronic periodontitis.

## Introduction

Periodontal disease is an inflammatory disease initiated by the colonization of microbial plaque while direct and indirect mechanisms of destruction, *i.e.,* bacterial tissue invasion and host response, are both involved (1, 2). In response to the bacterial plaque accumulation, the host tissues produce various inflammatory mediators and reactive oxygen species (ROS), which plays a major role in destroying the periodontal structures (2, 3). Interleukins, prostaglandins and TNF-α, key mediators involved in periodontal diseases (2, 4 and 5), have been widely studied as indicators for the presence or severity of periodontitis. The concentration of IL-1β in gingival crevicular fluid (GCF) is elevated in periodontally affected sites, and its tissue levels correlate with the severity of clinical periodontal disease (6, 7). 

One of the indirect mechanisms of inflammatory tissue destruction is the production of reactive oxygen species by neutrophils, including hydroxyl, oxygen peroxide (H2O2), and superoxide (O2) (8). Reactive oxygen species can cause serious damage to various intra- and extra-cellular macromolecules, including lipids, proteins, carbohydrates, and DNA (3), subsequently leading to periodontal attachment loss (3) and alveolar bone resorption (8). Elevated levels of these molecules have been found in GCF (8), saliva (9) and serum (10) in periodontally involved patients.

Most therapeutic strategies for periodontitis have focused on reducing bacterial challenges. Non-surgical periodontal treatment, although successful in many cases, maybe insufficient to remove bacterial plaque from deep pockets (11). Therefore, adjunctive systemic/topical antimicrobials or surgical interventions are used to improve the results of resistant cases (11). However, antibiotics may have adverse effects (12), and surgeries may lead to further attachment loss in shallow pockets (13). Maintenance of treatment outcomes with either method depends on adequate supportive therapy (14).

Host modulatory therapy is a new approach used in treating periodontal diseases aiming to damping destructive phases and enhancing protective and regenerative phases of the disease process (15). Antioxidant agents are developed to control various inflammatory diseases, which help control inflammation via decreasing ROS formation (3). A large number of drugs have been evaluated for their potential role in the management of periodontitis, but efforts are still in progress to find new medicines.

In recent years, a new registered herbal medicine named ANGIPARS (Semelil) has been developed. It is extracted from a plant called *Melilotus officinalis* (yellow sweet clover) which is a member of Fabaceae Legume family. Semelil is mainly composed of coumarin derivatives, flavonoids, and selenium that have anti-inflammatory, antioxidant, and angiogenic effects (16-18). Coumarin derivatives are effective against periodontal pathogens (19), to enhance extracellular matrix production by periodontal ligament stem cells (20), and to reduce the development of periodontitis in rats (21). Furthermore, promising results have been obtained by flavonoids in experimental and clinical research on periodontitis (22-24). Selenium, an essential micronutrient, also reveals antioxidant and anti-inflammatory properties (25), protecting periodontal tissues (26).

Preclinical and clinical studies showed the safety of the Semelil (27, 28). Several studies have proved its positive effects on the treatment of diseases like pressure and diabetic foot ulcers (29-31). Periodontal disease, with a destructive mechanism similar to diabetes mellitus (32), is reasonably assumed to benefit from host modulation therapy with Semelil. A previous animal study by authors evaluated the efficacy of Semelil on experimental periodontal disease and found it beneficial (33). This clinical study aims to assess the effect of Semelil as an adjunct to standard non-surgical treatment of periodontitis. 

## Experimental

*Patients population*

This research was designed as a preliminary parallel, triple-blind randomized controlled clinical trial and registered in the Iranian Registry of Clinical Trials (IRCT201307248898N2). Samples were selected from patients referred to the periodontics department, Tehran University of Medical Sciences. The study protocol was in accordance with the Helsinki Declaration of 1975, as revised in 2000. The study protocol was approved by the ethics committee of the Tehran University of Medical Sciences (reference number: 92-01-70-21279-99618). The inclusion criteria were systemically healthy patients with a minimum of 14 natural teeth and generalized moderate to severe chronic periodontitis, defined as the presence of ≥ 30% of sites with probing depth ≥ 5 mm, clinical attachment loss ≥ 3 mm, and bleeding on probing (34-36). Exclusion criteria were systemic disorders (*e.g.* diabetes mellitus, cancer, immune system disorders, bone metabolic disorders, diseases affecting healing potential, radiotherapy, and immunosuppressive therapies), smoking, pregnancy, lactation, consumption of systemic antibiotic during last two months, long-term consumption of non-steroid anti-inflammatory drugs, and any periodontal treatment in last year. From the authors, SA and MP examined and selected cases. Informed consent was obtained from patients eligible for participating in the study. 

*Sample size calculation*

According to previous study results (37), with an analysis power of 80% and a significance level of 0.05, a sample size of 20 was obtained for each group. The final sample size was chosen 22 per group to overcome the estimated 10% patient dropout.

*Treatment protocol*

All patients received periodontal phase I treatments, including oral hygiene instructions and non-surgical scaling and root planing. The initial treatment was performed by two periodontists. The procedure was repeated as often as necessary to reach the O’Leary plaque index of less than 30%. Patients were prohibited from consumption of any antibiotic during the study period. Then patients were randomized to test or control group, with an allocation ratio of 1:1, and received capsules of 100 mg ANGIPARS (ParsRoos Co., Tehran, Iran) or placebo, respectively, to take twice a day for three months. One researcher (MD) performed random allocation using a random number chart (designed by statistician) when the first patient was enrolled. The next patients were assigned according to the chart. Patients were blinded to the type of intervention they received. Patients were recalled and reinstructed for compliance with the drug consumption and oral hygiene measures every four weeks. Oral soft tissues were also examined for the potential drug-induced lesions.

*Examination parameters*

Clinical and laboratory parameters were measured at baseline and after completing the three months of adjunctive therapy. 

*Clinical Parameters*

Plaque index (38), modified gingival index (39), modified sulcular bleeding index (40), probing depth, and clinical attachment level were evaluated. All clinical measurements were performed by one calibrated periodontist (HA) blinded to the study groups. To be calibrated, the examiner measured the clinical parameters of 10 patients twice at two sessions with 24 h intervals. The intraexaminer agreement was 92%.

*Laboratory Parameters*

Interleukin-1β (IL-1β), lipid peroxidation (LPO), and 8-hydroxy-2-deoxyguanosine (8-OHdG) were evaluated in gingival crevicular fluid (GCF) samples taken from the patients. GCF sample of each patient was collected from the deepest periodontal pocket using a paper strip (PerioPaper Strips, Oraflow Inc, NY, USA). It was immediately centrifuged, frozen and kept at -80 ºC.

To measure LPO, the reaction of its products with the thiobarbituric acid (TBA) was utilized. TBA reacts with aldehydes such as malondialdehyde (MDA) – one of the lipid peroxidation products- in a high temperature and acidic environment to produce a complex with measurable pink color that has an absorbance at 532-535 nm. For this purpose, 25 mL trichloroacetic acid 20% was added to 0.05 mL of each sample, incubated at room temperature for 10 min, and then centrifuged at 1200 *×g* for 10 min. The precipitate was then solved in 0.025 mL of sulfuric acid 0.05 M and 0.02 ml of thiobarbituric acid 0.02%. The yielded solution was incubated for 30 min in a boiling water bath, cooled, and 0.04 mL of n-butanol was added to it. After generating a vortex solution, it was centrifuged at 1200 *×g* for 10 min. Finally, absorption of the supernatant was recorded at 532 nm by a spectrophotometer. Compared to the resultant resorption of the solutions with standard concentration, the concentration of the LPO products in the sample was calculated.

IL-1β and 8-OHdG were measured using a standard ELISA kit (Human IL-β ELISA kit 96t Diaclone, France; and Human 8-OHdG ELISA kit 96t zelbio, Germany, respectively). The absorption of the final product was measured at 450 nm for IL-1β and 412 nm for 8-OHdG.

*Statistical analysis*

The mean values of the clinical parameters were analyzed using point analysis and total analysis, while for the biochemical variables, only total analysis was performed. Repeated measure ANOVA was used to analyze changes in the indices with the intervention (drug/placebo) being considered as “between-subject factor” in either test or control groups. Data from biochemical factors were analyzed using one-way ANOVA, followed by Tukey. All analysis was performed by a statistician blinded to the test and control groups.

## Results

**Figure 1:**
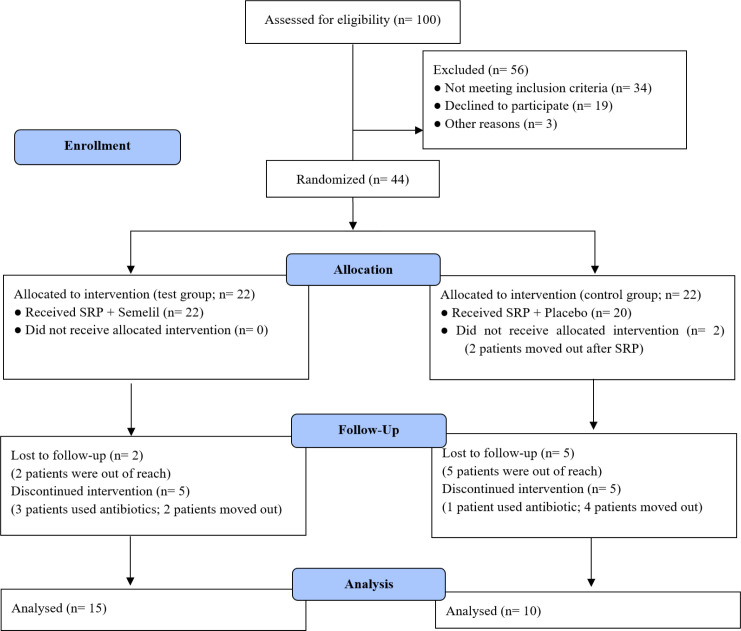
Consort flow diagram

From within 100 patients examined, 44 fulfilled the criteria and accepted to participate in the study. Only 25 patients followed the study protocol: 13 females and 12 males; 15 in the test group and 10 in the control group. Nineteen patients were excluded or lost to follow-up for various reasons (Figure 1). No adverse systemic and/or local effects were seen in test or control groups.

For data analysis, parameters measured in pockets with a depth of 5 mm or more were included. Two groups were statistically the same in baseline measurements.

*Clinical parameters*

All clinical variables were significantly improved in both groups after treatment. The modified gingival index showed the biggest changes among all the variables in both test and control groups. The differences between baseline and after-treatment clinical parameters in the test group treated with Semelil were significantly higher than the control group. These data and their analysis are presented in Table 1.

*Laboratory parameters*

After three months, biochemical parameters were significantly reduced in level in both test and control groups. Although the changes in the Semelil-treated cases were more prominent compared to the control samples, the difference between the groups was not statistically significant. Data related to laboratory parameters are available in Table 2.

## Discussion

Present research is the first human study evaluating the effect of Semelil on clinical and biochemical parameters of periodontal disease. The results showed that all clinical and biochemical parameters improved after treatment in both test and control groups. In periodontal pockets with a depth of 5 mm or more, the Semelil treatment showed significantly better outcomes in terms of plaque index, modified sulcus-bleeding index, modified gingival index, probing depth, and clinical attachment level compared to placebo. IL-1β, LPO, and 8-hydroxy-2-deoxyguanosine measured in the GCF from the deepest pocket of each patient were significantly reduced after treatment in both groups. This decrease was greater in the test group than in the control group, although the difference was not statistically significant.

Semelil was first introduced to treat diabetic foot ulcers and showed promising results (41, 42). In addition to benefits in treating some diseases, lack of toxicity (41, 43) led this herbal extract to be used widely. According to the proven anti-inflammatory and antioxidant properties (16, 17 and 44) of the ingredients of Semelil, it was hypothesized that the drug might modulate host response during periodontal diseases.

The presumed angiogenic effects of this medication (18) could also provoke periodontal regeneration (45). Reduced dental plaque score in the test group after treatment, although not significantly different from the control group, may reflect the effect of environmental changes on biofilm formation (46).

Various *in-vitro* and *in-vivo* studies have shown the ability of coumarin derivatives to suppress superoxide generation in leukocytes (47) and inhibit pathways of lipoxygenase (48, 49) and cyclooxygenase (48). Researches on flavonoids have also revealed their efficacy in clinical or experimentally induced periodontal inflammation to inhibit the production of ROS (50) and inflammatory cytokines (51), exert antimicrobial activity against periodontopathogens (52), and promote the proliferation of periodontal ligament stem cells (50). Consequently, the alveolar bone resorption (24, 53), probing depth, and bleeding on probing (23) have been reported to be reduced. The other ingredient, selenium also plays a major role in cellular antioxidant defensive systems (54). Various selenoproteins are involved in innate and adaptive immunity, and some have enzymatic antioxidant activities (26). The concentration of glutathione peroxidase, a plasma selenoprotein, is increased in the GCF samples taken from patients with periodontitis (55, 56). Reduced serum selenium has been associated with an increase in the prevalence of periodontitis (57). Based on the study by Navaei *et al.*, selenium-enriched medicines reduced oxidative stress, apoptosis and necrosis in lymphocytes exposed to chlorpyrifos, a toxic pesticide (58).

Similar to our findings, Mousavi *et al.* demonstrated that systemic administration of Semelil significantly reduced gingival IL-Iβ, 8-OHdG, and LPO in rats with periodontitis (33). On the other hand, in a study comparing Semelil and placebo in the treatment of diabetic patients (30), no change was seen in any of the oxidative stress markers except for deoxyguanosine which was significantly reduced in the test group. Hence, the authors concluded that positive therapeutic results of Semelil might be due to mechanisms other than antioxidant effects (30).

In an animal study on anethole (59), a flavonoid which is a major component of essential oils derived from aromatic plants, Moradi *et al*. concluded that the medicine could significantly reduce the blood levels of IL-1β and TNF-α. However, the effect size was not as large as the ketoprofen-treated group. These findings are in accordance with the results of the present study after Semelil administration, the IL-1β was significantly reduced in the GCF. Nevertheless, the difference was not statistically significant relative to the placebo group.

In a randomized controlled clinical trial, Hasani-Ranjbar *et al*. evaluated the effects of Semelil on markers of bone formation and bone resorption in diabetic patients (60). They measured bone alkaline phosphatase, osteocalcin, serum TNF-α, urine calcium, creatinine, and pyridinoline before and three months after treatment with 100 mg Semelil or placebo twice a day. The obtained results showed that only changes in urine creatinine were significantly different between the two groups. Therefore, the authors concluded that the Semelil has no positive or negative influence on bone remodeling.

The prescribed dose of the medication is, however, a determining factor. In a research on the effect of baicalin – a flavonoid compound with anti-inflammatory and antioxidant effects (61)- on ligature-induced periodontitis in rats, the concentration of 200 mg/kg of baicalin, and not 50 nor 100 mg/kg, significantly reduced the amount of alveolar bone loss (62). Similarly, the positive effects of Semelil, which also contains flavonoids, on bone loss may be only achievable in a particular dosage.

This study suffers from a small sample size and short-term follow-up. It is recommended to perform further controlled clinical trials with more participants and longer follow-up periods to obtain more accurate results. In addition, this study was performed on generalized moderate to severe chronic periodontitis. The effect size may be different for other disease extent and severity. Measurement of some of the other markers of tissue destruction may also be helpful in more precisely detecting the mechanism of action of Semelil in improving periodontal status.

**Table 1:** Clinical parameters before and after treatment.

**Group**	**Test**		**Control**		***p*** **-value**
**Number of pockets ≥5 mm**	220		138		
	**Baseline**	**After treatment**	**Baseline**	**After treatment**	
PI (mean ± SD)	1.75 ± 0.48	1.30 ± 0.65	1.96 ± 0.27	1.74 ± 0.50	0.070
	*p* < 0.001		*p* < 0.001		
MGI (mean ± SD)	2.57 ± 0.97	1.36 ± 0.73	1.92 ± 0.93	1.71 ± 0.84	0.004
	*p* < 0.001		*p* < 0.001		
MSBI (mean ± SD)	2.60 ± 0.59	1.98 ± 0.87	2.28 ± 0.61	2.00 ± 0.90	0.000
	*p* < 0.001		*p* < 0.001		
PD (mean ± SD) (mm)	5.57 ± 0.88	4.12 ± 1.67	5.21 ± 0.44	4.38 ± 1.33	0.000
	*p* < 0.001		*p* < 0.001		
CAL (mean ± SD) (mm)	5.79 ± 1.32	4.58 ± 1.94	5.22 ± 0.72	4.40 ± 1.26	0.025
	*p* < 0.001		*p* < 0.001		

**Table 2:** Biochemical parameters before and after treatment

**Group**	**Test**		**Control**		***p*** **-value**
**Number of patients**	15		10		
	**Baseline**	**After treatment**	**Baseline**	**After treatment**	
IL-1ß (mean ± SD) (pg/mL)	211.88 ± 23.89	164.03 ± 11.02	209.03 ± 13.51	175.02 ± 8.86	>0.05
	*p* < 0.001		*p* < 0.001		
LPO (mean ± SD) (µM)	19.41 ± 1.68	16.16 ± 2.10	19.14 ± 1.07	17.13 ± 0.63	>0.05
	*p* < 0.001		*p* < 0.001		
8-OHdG (mean ± SD) (pg/mL)	199.82 ± 19.06	159.07 ± 18.98	202.3 ± 10.14	171.73 ± 28.01	>0.05
	*p* < 0.001		*p* < 0.001		

## Conclusion

Systemic administration of Semelil with anti-inflammatory and antioxidant effects could be beneficial for treating chronic periodontitis. Randomized controlled clinical trials with a larger sample size and longer follow-ups are recommended.

## Conflict of interest

The authors declare no conflict of interest.
